# Trade-Offs between Avoidance of Noxious Electric Shock and Avoidance of Bright Light in Shore Crabs Are Consistent with Predictions of Pain

**DOI:** 10.3390/ani14050770

**Published:** 2024-02-29

**Authors:** Stuart Barr, Robert W. Elwood

**Affiliations:** School of Biological Sciences, Queen’s University, Belfast BT9 5DL, UK; s_barr@hotmail.com

**Keywords:** pain, decapod, *Carcinus maenas*, trade-off, avoidance, learning, anxiety

## Abstract

**Simple Summary:**

The idea that invertebrates feel pain is often dismissed because of claims that their responses to noxious stimuli are simply reflexes. Reflexes do not require higher-order processing, but that is required for pain experience. One way to test this claim is to ask if animals only respond to the noxious stimulus or if that response can be influenced by other motivations. Here, we report that shore crabs that enter a dark shelter from a light area and receive a shock are subsequently less likely to use that shelter if the light level is low and the shock level high. They trade their requirement to avoid bright light for the avoidance of the noxious shock. They learn to avoid the shelter and show signs of anxiety when deciding to enter the shelter to escape the light. These observations demonstrate that the responses to shock are not reflexes but are decisions that should improve the outcome better than a reflex alone. This balancing of needs is a key expectation of pain, and the results are consistent with the idea that crabs feel pain.

**Abstract:**

The suggestion that decapod crustaceans might experience pain has been dismissed by some authors who claim decapods only respond to noxious stimuli by nociceptive reflexes. Because reflexes do not require complex neuronal processing, but pain does, demonstrating reflex responses to noxious stimuli would not support the case for pain. Here, we report an experiment in which shore crabs are repeatedly placed in a light area (20 trials), but the animals can avoid the light by moving to a dark shelter. However, some crabs received an electric shock of 6 or 12 volts each time they entered the shelter. Those receiving either level of shock swiftly reduced their use of shelters and remained in the light. However, the magnitude of shelter avoidance was influenced by the brightness of the arena and the intensity of the shock. Shelter use was subsequently reduced to a greater extent if the shock level was high and the light intensity low. That is, crabs traded their avoidance of shock for their avoidance of bright light. Further, these animals showed avoidance learning and demonstrated activities suggesting anxiety, such as contact with the tank wall in the light area and increased latency to enter shelters when making the decision to enter the shelter if they had received shock in earlier trials. These results fulfil three key behavioural criteria for pain and, thus, are consistent with the idea that decapods can experience pain.

## 1. Introduction

Experiments on possible pain in invertebrates tested various criteria [1,2,3,4], and if these criteria are fulfilled, the data are said to be consistent with the idea of pain [1]. However, no single criterion provides an all-or-none test for the existence of a subjective sense of pain within a non-human taxon; rather, we must rely on an accumulation of evidence that supports the hypothesis that pain occurs [2,4,5]. Key expectations of pain are that it enables long-term changes in behaviour so that future injury or tissue damage is reduced and recovery from the current injury is enhanced [6]. These behavioural changes are expected to be more complex than nociceptive reflexes [2]. Here, we describe a single experiment that investigates several commonly accepted criteria. 

One key criterion is that we would expect to see optimal choices in behaviour by which the avoidance of the noxious stimulus is traded-off for other motivational requirements. A response that is purely mediated by a nociceptive reflex should be the same regardless of other motivational priorities. That is, we would expect to see the same reflex avoidance of a noxious stimulus in an animal that is hungry or satiated, even if food is present. However, if we see variation in the response to noxious stimuli that is dependent upon other motivational requirements, then there must be some higher-level interaction between competing motivational systems [7]. 

In birds, for example, we see that motivational changes in the chicken (*Gallus gallus* var *domesticus*) can alter the bird’s attention and significantly alter pain-related behaviour [8]. An injection of sodium urate into the ankle joint of the chicken mimics acute gouty arthritis and elicits pain-coping behaviour, such as one-legged standing, limping, and sitting [9]. However, an increase in feeding motivation, produced by a 16 h deprivation, resulted in a prolonged period of feeding activity and almost total analgesia to the severe tonic pain of sodium urate arthritis [10]. Similarly, we see that goldfish (*Carassius auratus)* deprived of food are less likely to respond to an electric shock in a feeding area than those that are not deprived of food [11]. Bees also show trade-offs between feeding and the avoidance of high temperatures, as shown by less avoidance of heated feeders when a high-value food source might only be obtained from that feeder [12]. 

Studies on decapods tended to focus on trade-offs between the avoidance of a noxious stimulus and requirements other than food. For example, hermit crabs were more likely to abandon an unpreferred species of shell, compared to a preferred species, when exposed to an electric shock within the shell [13], and they abandoned less-preferred species at a lower level of shock than they did from more-preferred species [14]. Further, hermit crabs were less likely to abandon shells when shocked if the odour of a predator was present [15]. 

Here, we examine potential trade-offs in shore crabs, *Carcinus maenas*, between the avoidance of brightly lit areas and the avoidance of electric shock within a dark shelter. In each trial, the crab can decide to enter the dark shelter and receive a shock, or it can remain in an arena that is illuminated. In the littoral zone, shore crabs hide under rocks to avoid visual predators, such as gulls, but may emerge at night to forage [16]. In the experiment, we vary the illumination between one that is bright and one that has a much lower illumination and examine how illumination affects the decision to enter the shelter. If the light intensity has no influence on the response to electric shock, the latter may be likened to a nociceptive reflex, but if there is a trade-off, then a reflex can be ruled out [2]. 

While the main aim of the current study is to examine trade-offs, we examine aspects of behaviour that relate to other criteria. For example, a key function of pain is to enable the animal to avoid noxious stimuli [1,5], and avoidance learning has been demonstrated in shore crabs [17]. Here, we ask if there is evidence of a shift in the use of shelters when they are associated with a noxious stimulus. We further examine activities that might indicate anxiety [18] when faced with a choice of remaining in bright light or moving into a dark area that might result in shock. 

These criteria are tested in an experiment in which shore crabs, *C. maenas*, are presented repeatedly with the choice of entering a dark shelter, and for some of the crabs, that decision results in an electric shock. We vary the value of the shelter by varying the level of light outside of the shelter and the cost of entering the shelter by varying the level of shock. 

## 2. Materials and Methods

### 2.1. Collection and Experimental Treatments

European shore crabs (*C. maenas*, carapace width = 5–8 cm) of both sexes were collected using baited crab pots from Bar Hall Bay, Strangford Lough (OS; J 617464, Portaferry, UK), between February and April 2008. Only fully intact crabs, with hardened exoskeletons and not carrying eggs, were transported to Queen’s University, Belfast. The crabs were housed in plastic tanks (76 × 38 × 17 cm), fitted with a lid, and filled with aerated seawater to a depth of 3 cm, and seaweed was provided for shelter (*Ascophyllum nodosum*) in a cold room maintained at 11–13 °C with a twelve-hour light/dark regime. The light level within the tank during the light period would depend on where the animal was positioned with respect to the seaweed provided. Seawater was changed every 2 days after the crabs were fed fresh mackerel pieces. Crabs were used within two weeks of collection.

A tank was prepared (30 × 19 × 20 cm) by dividing it into a dark shelter and a light area. The shelter (14 × 19 × 2.5 cm) consisted of a sheet of corrugated plastic placed horizontally on supports at one end of the tank. The shelter was made dark by attaching black plastic to the shelter roof and the tank walls immediately above the shelter. The entrance to the shelter was 2.5 cm high, and the shelter could be lifted to facilitate removal of the crab at the end of trials. The light area of the tank (16 × 19 × 20 cm) was illuminated either by a 12 W bulb (180 Lux) or a 100 W bulb (2060 Lux). Light intensity was measured by Sekonic Model L-608 Cine (Super Zoom Master, Sekonic, Tokyo, Japan). Seawater was added to a depth of 1 cm before placing the tank under the predetermined light bulb in an observation chamber (71 × 36.4 × 39.2 cm) behind a one-way mirror so the observer could view directly into the shelter without disturbing the animal. 

Each subject was brought from the cold room to the observation room, and two wires (7/0.1 mm insulated wires with insulation removed at the ends) were looped over and tightened around each hind leg at the joint between body and limb. The other ends of the wires were connected to a Grass S9 electric stimulator (Grass Instrument Company, West Warwick, RI, USA), which generated the electric impulses. The crab was placed into the light area, facing towards the shelter, and the observation commenced. If it moved into the shelter, it received a predetermined single shock of 6 or 12 volts (180 Hz, 200 ms); or no shock if it was in a no shock group. If the crab remained in the light area during the 2 min trial, it did not receive a shock. After each trial, the crab was lifted and placed (or replaced) in the light area without a break between trials. Each crab was subjected to 20 trials. Any crab that autotomised a limb was replaced by a new crab, starting with trial 1. 

For each trial, we recorded if the crab entered the shelter, the time taken to enter the shelter, the time spent in the light area, the time spent in the shelter, and the time at which the crab left the shelter. The escape response, comprising running immediately following shock and exits from the shelter, was recorded. Contact with the sides of the tank in the light area was also recorded. This involved the crab pressing against the sides of the tank with its chelipeds, backing into the tank sides, or, in some cases, attempting to climb the tank wall. 

The crabs were randomly assigned (by drawing tokens from a bag) to one of the six experimental groups (*n* = 10 per group): (i) 0 V, 12 W; (ii) 0 V, 100 W; (iii) 6 V, 12 W; (iv) 6 V, 100 W; (v) 12 V, 12 W; and (vi) 12 V, 100 W. All shocks had a frequency of 180 Hz and duration of 200 ms. After each experiment, the crabs were returned to the cold room and housed in tanks separated from the unused crabs before being released back in Strangford Lough. Both sexes were used in experiments and allocated randomly. However, most subjects were male. The small number of females in each experimental group precluded analysis of sex effects on behaviour. 

### 2.2. Statistical Methods

The escape response was represented as a percentage of the number of entries that resulted in immediate running and analysed using a two-way ANOVA (factor 1—light intensity; factor 2—voltage). Following shock, the incidence of crabs leaving the shelter was analysed using Fisher’s exact tests for the effect of both light and voltage.

A three-way ANOVA, with trials as a repeated measure (four blocks of 5 trials) and light intensity and voltage as independent factors, examined the number of trials in which the crab entered the shelter. The number of entries in the fourth quarter (final 5 trials) was analysed by two-way ANOVA (factor 1—light intensity; factor 2—voltage) to determine the effects of the prior experience of repeated testing. 

The mean latency to enter the shelter was calculated for each crab, and in each case, the 1st trial was excluded because the crab was not previously subject to shock on that occasion, as were occasions when the shelter was not entered. The mean latency to enter the shelter was analysed using a three-way ANOVA, with time as a repeated measure (mean for trials 2–10 and 11–20) and light intensity and voltage as independent factors. 

Contact with the tank sides in the light area was calculated as a percentage of the average time spent in the light area and analysed with a two-way ANOVA (factor 1—light intensity; factor 2—voltage). Data for contact with the tank sides in the light area were transformed to log(x + 1).

### 2.3. Ethical Considerations

This experiment was conducted in 2008 when there was little or no support for the idea of pain in decapod crustaceans. There were no legal restrictions on experiments on this group of animals. Nevertheless, we kept the number of animals low in each experimental group (*n* = 10 per group), and we kept the level of shock as low as possible, and no shock was given in two of the six groups. The results of similar experiments, published between 2008 and 2017 [2], provided evidence to suggest the idea of pain in decapods, and the legal situation for decapods has subsequently changed within the United Kingdom, which now recognizes that these animals are sentient. Despite this, there has been no change in the UK legal requirements for research on decapods, and thus, the experiment is fully compliant with current UK regulations. Nevertheless, we suggest that researchers should take the potential sentience of these animals into account when designing future studies rather than waiting for legal change and refer to guidelines on the use of decapods [19].

## 3. Results

### 3.1. Immediate Response to Shock

**Figure 1:**
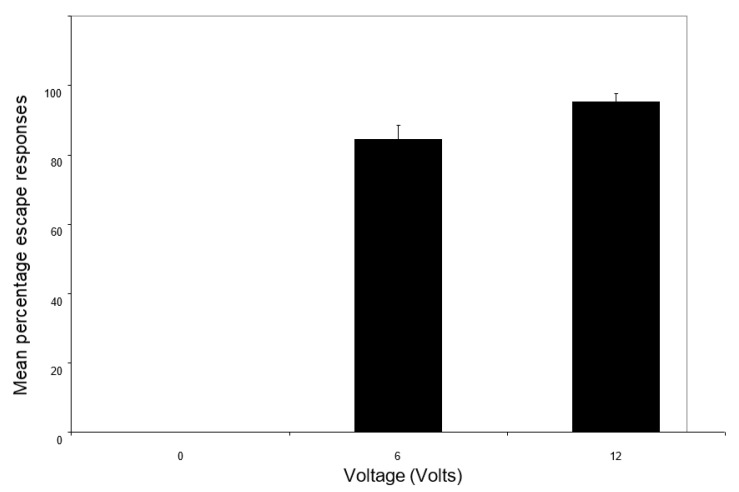
The effect of shock level on the mean percentage of escape responses (±SE) observed upon shelter entry.

There was no significant effect of light intensity on the percentage of trials in which animals ran after a shock within the shelter (F_1,54_ = 2.48; *p* = 0.12); however, voltage did have an effect because crabs only showed this escape response when they were shocked (F_2,54_ = 378.66; *p* < 0.0001; Figure 1). There was no interaction between light and voltage on the percentage of trials with escape responses (F_2,54_ = 1.9; *p* = 0.16). 

Crabs that received a shock of 12 V were significantly more likely to exit the shelter compared to those that received 6 V (6/20 vs. 0/20; Fisher exact test *p* = 0.02) or no shock (6/20 vs. 0/20; Fisher exact test *p* = 0.02). However, crabs exposed to low light did not exit the shelter significantly more than those experiencing high light outside the shelter (4/30 for 12 W vs. 2/30 for 100 W; Fisher exact test *p* = 0.39).

### 3.2. Decisions to Enter the Shelter

In the first trial, no crab received a shock prior to entering the shelter; thus, a shock could not affect the decision on that trial. Most crabs entered the shelter in the first trial, but this was not influenced by the light level, with 27/30 entering from low light and 30/30 entering from bright light (Fisher exact test *p* = 0.237).

**Figure 2:**
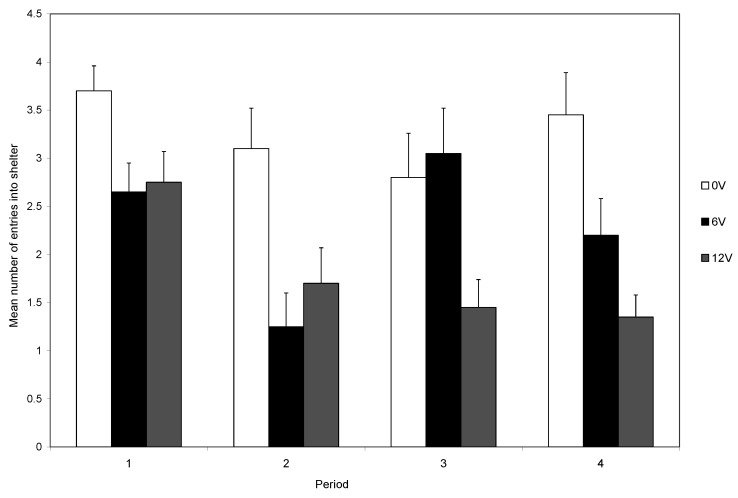
The effect of voltage on the mean number of entries (±SE) into the shelter over the four sets of five trials.

To examine shelter entries over the course of the experiment, we used a three-way ANOVA with light level and shock level as independent factors and time (four blocks of five trials) as a repeated measure. The three-way interaction was not significant (F_6,162_ = 2.026, *p =* 0.651). However, the two-way interaction between voltage and time was significant (F_6,162_ = 5.362, *p* < 0.0001; Figure 2). Crabs that were not shocked maintained a high number of entries over the four sets of trials, whereas crabs exposed to 12 V showed a decrease over the four blocks of trials, and crabs exposed to 6 V showed a more variable pattern. 

**Figure 3:**
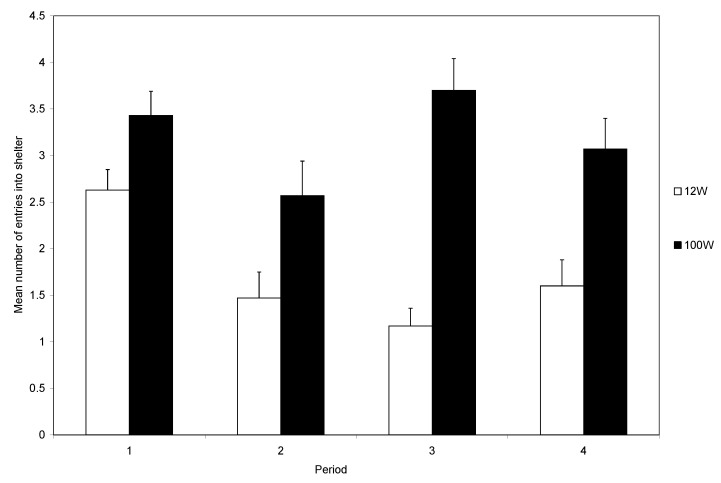
The interaction effect between light intensity and time on the mean number of entries (±SE) into the shelter over four sets of five trials.

The two-way interaction between light intensity and time was also significant (F_3,162_ = 7.363, *p =* 0.0001; Figure 3). Crabs in the high light intensity maintained a high rate of shelter entry, whereas those in the lower light showed a marked decrease in shelter entry.

**Figure 4:**
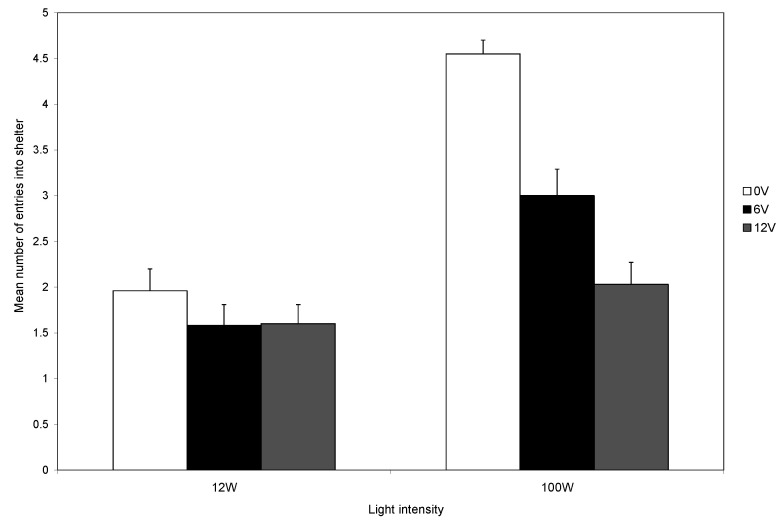
The effect of light intensity and voltage on the mean number of entries (±SE) into the shelter expressed as the average number per block of 5 trials.

The interaction effect between light intensity and voltage on the number of entries was significant (F_2,162_ = 6.054; *p* < 0.05; Figure 4). This was because, at low light intensity, crabs had a low level of shelter entry regardless of the level of shock, whereas in high light intensity, there was a marked effect of shock level. The three main effects were all significant. Crabs were more likely to enter the shelter when the light intensity was high (F_1,162_ = 34.14; *p* < 0.0001) and when the voltage was low (F_2,162_ = 11.433; *p* < 0.0001), and there was a decrease in the number of entries following the first five trials (F_3,162_ = 9.298, *p* < 0.0001). 

**Figure 5:**
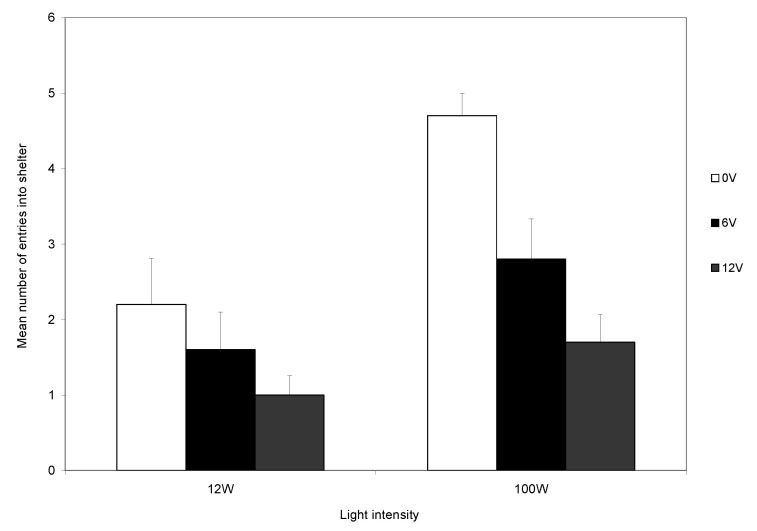
The effect of light intensity and voltage on the mean number of entries (±SE) during the final five trials.

In the final quarter, the number of times the crabs entered the shelter was higher in high light intensity (F_1,54_ = 16.16, *p* < 0.001), and there was a significant effect of voltage on the number of entries into the shelter (F_1,54_ = 11.18; *p* < 0.0001), with the number decreasing as the voltage increased. There was no significant interaction effect (F_1,54_ = 2.16; *p* = 0.125; Figure 5).

### 3.3. Activities in the Light Area

**Figure 6:**
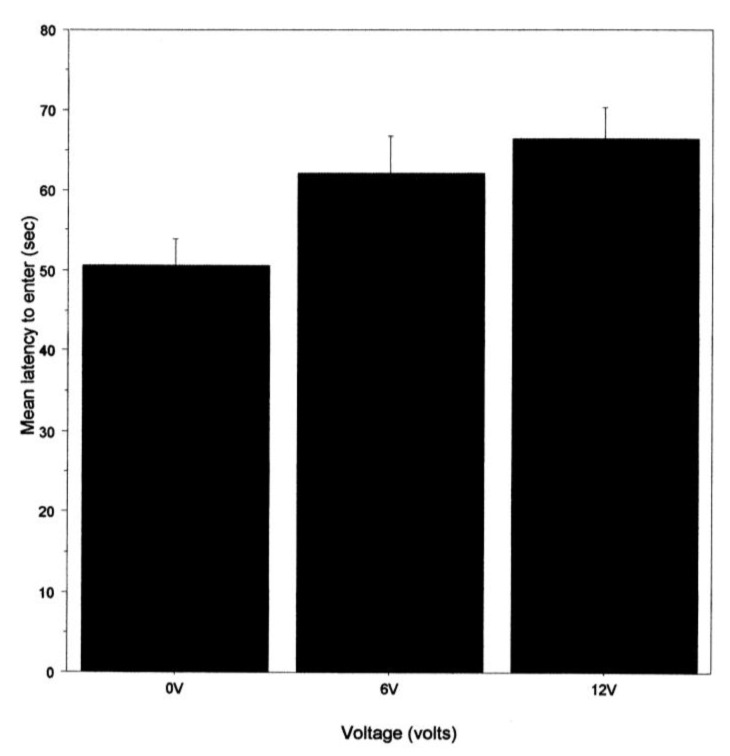
The effect of voltage on the mean latency (±SE) to enter the shelter for those that entered on trials 2–20.

We used a three-way ANOVA to explore if there was variation in the mean latency to enter the shelter (just for animals that entered) between the first and second halves of the experiment with respect to the light and shock intensity. This demonstrated that latency increased with shock intensity (F_2,45_ = 3.26; *p* < 0.05; Figure 6) but did not differ between light intensities (F_1,45_ = 1.84; *p* = 0.18) or between the two periods of testing (F_1,45_ = 0.27, *p =* 0.6). None of the interaction terms were significant. 

**Figure 7:**
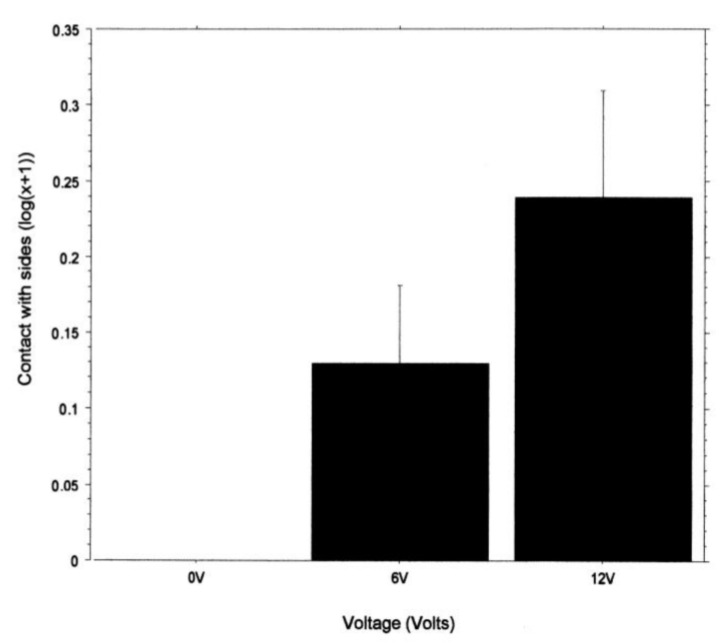
The effect of voltage on the mean time spent (±SE) in contact with the edge of the light area, represented as a percentage of the average time spent in the light area (log (x + 1)).

Time spent in contact with the sides of the light area (as a proportion of total time in the light) was not significantly affected by light intensity (F_1, 54_ = 0.402; *p* = 0.53); however, it was significantly affected by voltage (F_2, 54_ = 5.57; *p* < 0.01; Figure 7), with edge contact increasing as the voltage increased. There was no significant interaction effect (F_2, 54_ = 1.26; *p* = 0.29).

## 4. Discussion

Crabs that were given shocks received those shocks as the crab entered the shelter. These shocks usually elicited immediate running, which involved brief, rapid locomotion that usually took the crab deeper into the shelter and appeared to be a nociceptive reflex response. Because this was never seen in crabs that did not receive a shock, we interpreted the running as an attempt to escape from the noxious stimulus. That is, the stimulus was aversive. Those receiving the 12 V shock were only slightly more likely to show this escape response compared to those receiving the 6 V, so both levels of shock appeared to be aversive. However, only those receiving the 12 V shock emerged from the shelter, so it is reasonable to conclude that although both shock levels were aversive, the greater shock level was the more aversive. By leaving the shelter, the crab is giving up a resource that is important for survival. In the natural situation, a crab that leaves its shelter from under a rock during daylight will be at risk from visual predators [16]. 

The aversive nature of shock is further demonstrated by the subsequent reduced entry to the shelter over the twenty trials. Those that did not receive a shock continued to use shelters, but those that were shocked showed a marked decline in shelter entry, and this decline was particularly marked if the higher-level shock was given (Figure 2). Thus, electric shock is aversive to decapods, as was shown in previous studies, and shock provides a reliable, repeatable, noxious stimulus [14,17,20]. 

During the first trial, most crabs (95%) entered the shelter, and the number entering was not influenced by the light intensity. At the start of subsequent trials, each crab was placed back in the light area. Those that had received a shock could avoid another shock only if they remained in the light area. It was clear that the level of light influenced the decision to remain in the arena rather than enter the shelter during the remainder of the experiment (Figure 4). Those in bright light maintained a high rate of shelter use, whereas crabs in dull light rapidly reduced their use of shelters (Figure 3), indicating that the response to shock is influenced by motivations other than shock avoidance. The trade-off is seen clearly during the final five trials and the level of shock influences the use of the shelter in both light levels, although the reduction was greater in low than in high light (Figure 5). However, crabs in low light and that did not receive shocks also reduced their use of the shelter (Figure 5). 

All crabs were caught at the end of each trial, and if they were in the shelter, they were removed. *C. maenas* shows stress responses to experimental handling [21], and thus, the handling may dissuade crabs from using the shelter, even though they were handled at the end of all trials, whether they used the shelter or not. It is possible that being taken out of the dark shelter is more stressful than being picked up from the open light arena.

This trade-off is like those seen in hermit crabs, which were less likely to give up their shell (their moveable shelter) if the odour of a predator was present [15] or if they occupied a good quality shell [13,14], and in bees, which more readily gave up feeding from heated feeders if another good quality food source was available [12]. In each case, the value the animal places on one resource influences how it responds to a noxious stimulus, which demonstrates that the response to the noxious stimulus is not just a nociceptive reflex; rather, it involves the integration of different sources of information [2]. In the case of hermit crabs, the value of the shell could be because of variation in species and the crabs showing strong species preferences [13,14] or because some other factor, such as cues about a predator, changes the apparent value of the shell [15]. Studies on animal fights refer to these different types of resource value as objective and subjective, respectively [22]. Further, studies on aggression show that the fight costs that are accepted before a contestant quits are traded off against the resource value, suggesting that trade-offs are a common feature when animals make decisions [7]. In the present study, the shore crabs had the same standard shelter, but the potential cost of leaving it and moving to bright light is potentially higher than moving to dull light. Bright light seems to be equivalent to the odour of a predator, which is a subjective value. Because these trade-offs go beyond a nociceptive reflex, it is consistent with the idea of pain [2,4]. It shows that the response to the stimulus is flexible and enables the avoidance response to be more fitness-oriented than a reflex. A reflex should be the same regardless of other motivational requirements, whereas a decision that takes other motivational factors into account is a key predictor of pain because pain provides a better outcome in terms of fitness than a reflex [23].

Crabs exposed to shocks in the present study showed a swift reduction in the use of shelters, especially in the 12 v groups. That is, the behaviour changed because of the experience of shock, and this was noted in other experiments on place avoidance (Denti et al. 1988) [24], where a crab could choose between two locations, one of which was paired with shock, or discrimination avoidance, where a crab could choose between a bright arena and two dark shelters when only one of the latter was paired with shock [24]. Pain is thought to facilitate rapid avoidance learning, and thus, learning is a key criterion of pain [1,2,4]. The avoidance learning reported here fulfils another criterion for pain and is, thus, consistent with the idea of pain in decapods.

We would anticipate that exiting the shelter to escape the noxious stimulus would also show trade-offs with the light intensity. That is, the crab should be more likely to leave if the external light intensity is low. However, although more exits were seen when the light was low, the numbers exiting were small and not statistically significant. To investigate trade-offs when leaving the shelter, we need an experiment that induces more shelter exits. This could involve increasing the voltage or giving repeated shocks in each trial rather than just the one used here. When hermit crabs were shown to trade-off avoidance of electric shock for the avoidance of predator odours [15] or to keep high-quality shells, repeated shocks were used [13,14].

To examine if there might be indications of anxiety [18] about entering a shelter that was associated with a noxious stimulus, we recorded the latency to enter the shelter. This was not influenced by the light intensity in the arena but was by the prior shock level. That is, latency was shortest when there was no shock and longest for the 12 v group. This is like a previous study that seemed to show some hesitation to enter a shelter in which a shock was delivered [20], but, as with that previous study, we cannot exclude the possibility that a shock toward the fifth leg reduces the ability to walk. We note, however, that noxious chemicals applied to the eyes or antennae of two species of prawns also increased the latency in the initiation of walking [25,26], and this is unlikely to be caused by reduced walking ability. We also examined contact with the walls of the arena as a percentage of the time the crabs were in the arena. This showed that only crabs in the shock groups contacted the wall. The contact often involved the animal pressing into the sides of the tank with its chelipeds, backing into the tank sides, or, in some cases, attempting to climb the tank wall. These activities of shocked crabs are consistent with attempting to find an alternative hiding place, whereas the non-shocked crabs simply went into the shelter. This hyperactivity was observed in the crab *C. granulatus* when shocked in one compartment of a tank [27]. This suggests that the increased latency to enter the shelter is not due to decreased mobility but may represent a hesitation to move into a desired shelter because it is associated with shock. The finding that shocked crabs engage in alternative activities might account for the increased latency to enter the shelter and is consistent with the crabs apparently showing signs of anxiety when faced with a choice of remaining in the light or going into a dark shelter when the latter will produce a shock. Such shifts in behaviour were shown to improve survival from predatory fish in squid [28] and amphipods [29] and, thus, support the suggestion that pain must improve fitness beyond that achieved with nociceptive reflexes [1,23].

## 5. Conclusions

We showed that shore crabs will trade the avoidance of electric shock for the avoidance of bright light. Second, we showed that crabs reduce their use of a shelter associated with shock and, hence, show place avoidance. Third, crabs appear to be hesitant about entering a shelter associated with shock and engage in alternative activities to avoid the shock, which suggests anxiety. These observations fulfil three key criteria expected of pain, and thus, the present findings are consistent with the idea of pain. Apart from the escape running response when the shock was delivered, it is difficult to imagine how these observations might be mediated by nociceptive reflexes. However, that does not mean that we can prove precisely what the animal is feeling. Nevertheless, the present observations, and those of numerous other experiments, add to the idea of pain in decapods. For example, we saw a directed rubbing of the site of noxious stimulus application [25,30,31,32], swift avoidance learning [17,33], long-term changes in behaviour [13,31], anxiety [18,34], giving up vital resources to escape [20], physiological changes [18,34,35,36], in addition to trade-offs noted above. With the increasing number of studies that provide data consistent with expectations of pain, there was an increasing acceptance that pain is possible or even likely. The United Kingdom of Great Britain and Northern Ireland has recently recognized that these animals are sentient by passing The Animal Welfare (Sentience) Act (2022) [37]. With this increased acceptance that pain is likely, we should improve welfare standards regarding how vast numbers of decapods are used in the food industry and protect them from extreme practices, such as dismembering or boiling them without first killing or stunning them [4,37,38]. Further, we urge that attempts to avoid or reduce potential suffering should be taken when decapods are used in research [19,39].

## Data Availability

The data presented in this study are available on request from the corresponding author. The data are not publicly available due to it being on an old program.
